# Community-led research for reproductive justice: Exploring the SisterLove Georgia Medication Abortion project

**DOI:** 10.3389/fgwh.2022.969182

**Published:** 2022-08-12

**Authors:** Elizabeth A. Mosley, Sequoia Ayala, Zainab Jah, Tiffany Hailstorks, Dázon Dixon Diallo, Natalie Hernandez, Kwajelyn Jackson, Indya Hairston, Kelli S. Hall

**Affiliations:** ^1^Center for Reproductive Health Research in the Southeast (RISE), Emory University School of Public Health, Atlanta, GA, United States; ^2^SisterLove, Inc., Atlanta, GA, United States; ^3^National Birth Equity Collaborative, Washington, DC, United States; ^4^Department of Gynecology and Obstetrics, Emory University School of Medicine, Atlanta, GA, United States; ^5^Center for Maternal Health Equity, Morehouse School of Medicine, Atlanta, GA, United States; ^6^Feminist Women's Health Center, Atlanta, GA, United States; ^7^Department of Population and Family Health, Columbia University Mailman School of Public Health, New York, NY, United States

**Keywords:** community-led research, community-based participatory research, reproductive justice, abortion, reproductive coercion, medical distrust

## Abstract

**Introduction:**

While reproductive injustice indicators are improving globally, they are worsening in the United States particularly for Black and other marginalized communities. Eugenics and obstetric violence against low-income and communities of color create well-founded distrust of sexual and reproductive health (SRH). Transformational, reparative ways of conducting SRH research are needed.

**Proposed principles of community-led research for reproductive justice:**

Drawing on our collective experience as reproductive justice leaders, SRH researchers, and clinicians, we propose the following principles of community-led research for reproductive justice: 1) Center the marginalized community members most affected by SRH inequities as leaders of research; 2) Facilitate equitable, collaborative partnership through all phases of SRH research; 3) Honor multiple ways of knowing (experiential, cultural, empirical) for knowledge justice and cross-directional learning across the team; 4) Build on strengths (not deficits) within the community; 5) Implement the tenets of reproductive justice including structural-level analysis and the human rights framework; 6) Prioritize disseminating useful findings to community members first then to other audiences; 7) Take action to address social and reproductive injustices.

**SisterLove's community-led georgia medication abortion project:**

We offer the community-led Georgia Medication Abortion (GAMA) Project by reproductive justice organization SisterLove from 2018–2022 as a case study to demonstrate these principles along with the strengths and challenges of reproductive justice research.

**Discussion:**

Community-led reproductive justice research offers innovative and transformational methods for truly advancing SRH in an era of increasing policy restrictions and decreasing access to care. Yet existing funding, research administrative, and publishing systems will require structural change.

## Introduction

### Current landscape of U.S. reproductive health

While reproductive health and justice indicators are improving globally, they are worsening in the United States (1–3). For every 100,000 U.S. live births, there are 17 maternal deaths—over double that of other high-income nations (1). Non-Hispanic Black women die from pregnancy-related causes at 3 to 4 times the rate of non-Hispanic white women, and rates of maternal mortality are particularly high in the South (3). In Georgia, the statewide rate is 26 maternal deaths per 100,000 live births, but that is 17.4 for white Georgians and 47 for Black Georgians (4). Among high income nations, the United States also has the second-worst supply of midwives and obstetrician-gynecologists (only 15 per 1,000 live births) (1). Maternity care deserts are particularly dire across the rural South. In Georgia, an estimated 50% of counties have no OBGYN services and 40% of the state's rural labor and delivery units have closed since 1994 (5).

Despite the worsening maternal health crisis, conservative U.S. leaders continue to dismantle protections for abortion care. The Supreme Court of the United States is posed to overturn *Roe v. Wade* this month, thus eliminating federal abortion protection and allowing states to regulate or outlaw abortion (6, 7). Since *Roe v. Wade* in 1973, federal (e.g., the Hyde Amendment that prohibits federal funding to cover abortion for public insurance patients) and state-level (e.g., gestational age limits) policies have restricted access, particularly for people of color, low-income communities, and young people (8, 9). This creates treacherous double binds, particularly for Black and low-income people, who have the highest risk of maternal mortality and highest rates of abortion demand (due to poverty and lower access to contraception), but the worst barriers to safe abortion care (10).

Moreover, given historical and ongoing eugenics and obstetric violence against low-income and communities of color, they have widespread and well-founded medical distrust (11–14). Modern U.S. obstetric and gynecological techniques were developed on Black enslaved women, who did not consent and were given no anesthesia (15). The U.S. eugenics movement from 1900 to the 1970s—based in racist, classist, and ableist “science” and aimed at eliminating “social ills” through population control—forcibly sterilized over 60,000 individuals (14). Most of them were living with disabilities, from low-income backgrounds, or people of color. In fact, incarcerated and detained Latina women in the U.S. continue to be forcibly sterilized—most recently in 2020 at a detention center in rural Georgia (16, 17). And early U.S. contraceptive trials were conducted on Latinx Puerto Rican women, which resulted in infertility, reproductive cancers, and other health consequences (18).

Given this context, we need transformational, reparative ways of conducting SRH research that advance a human rights framework for reproductive justice—in the United States and globally.

### Reproductive justice framework and movement

Reproductive justice is an intersectional feminist theoretical framework, community organizing strategy, and social movement that is well-suited for transformational and reparative research and praxis (19, 20). It was first developed in the 1980s by Founding Mothers of color (including co-author Dázon Dixon Diallo^1^), who were marginalized by the white-led reproductive rights movement both in the United States and in global forums (such as the 1994 United Nations International Conference on Population and Development in Cairo) that focused almost exclusively on contraceptive and abortion access (19, 20). Grounded in the comprehensive human rights framework (21–24), reproductive justice leaders call for an expansive approach to sexual and reproductive autonomy that centers the experiences of Black, Brown, indigenous, immigrant, LGBTQ, and other marginalized communities (25–27). It uses an intersectional framework, based on Kimberlé Crenshaw (28) and other scholars' work, that emphasizes how social systems of oppression—such as racism, sexism, ethnocentrism, capitalism, colonialism, ableism, heterosexism—*intersect* and create unique experiences of social disadvantage and privilege. In other words, the SRH struggles of high-income white cisgender women (e.g., legal access to abortion and contraception) are distinct from the SRH struggles of low-income queer women of color (e.g., forced sterilization, low wages, employment and housing discrimination). Because of these intersectional oppressions and identities, true reproductive autonomy cannot be achieved for all without fulfillment of these conditions:

- The human right to have children.
- The human right not to have children.
- The human right to parent one's children in safe, supportive environments.
- Free of reproductive coercion or violence.

Today, the reproductive justice movement has grown to include hundreds of community-based organizations across the United States and internationally, who are leading grassroots social movements for voting rights, safe working and housing conditions, healthcare access, and more.

### Community-engaged and participatory action research

Traditional research approaches, where academic researchers are upheld as experts and community members most affected by negative health outcomes are exploited for data, are antithetical to reproductive justice and counter-productive for achieving SRH equity. Conversely, reproductive justice leaders utilize a research justice (29) approach, wherein Black, indigenous, and other marginalized community members are equally valued as experts. A research justice approach challenges the traditional knowledge hierarchy, where mainstream so-called scientific knowledge is privileged (has greater political power) over other ways of knowing: experiential, cultural, and spiritual knowledge. This is well aligned with community-based participatory research methods and similar approaches like community-engaged research and participatory action research (30, 31). Community-based participatory research is guided by these principles:

- Acknowledge community as a unit of identity.
- Build on community strengths and resources.
- Facilitate collaborative, equitable partnership in all phases of the research.
- Foster co-learning among all partners.
- Integrate and achieve a balance between knowledge generation and intervention.
- Focus on local relevance and multi-level ecological perspectives.
- Involve systems development using cyclical, iterative processes.
- Disseminate results to all partners, who are all involved in dissemination efforts.
- Engage in long-term process and commitment to sustainability (31).

## Proposed principles of community-led research for reproductive justice

Rooted in the reproductive justice and community-engaged research approaches and drawing from our collective experience as reproductive justice advocates, community-engaged SRH researchers, and clinicians, we propose these corresponding yet more specific principles of community-led research for reproductive justice (see Table 1):

- Center the marginalized community members most affected by SRH inequities as leaders of research
- Facilitate equitable, collaborative partnership through all phases of SRH research
- Honor multiple ways of knowing (experiential, cultural, empirical) for knowledge justice and cross-directional learning across the team
- Build on strengths (not deficits) within the community
- Implement the tenets of reproductive justice including structural-level analysis and the human rights framework
- Prioritize disseminating useful findings to community members first then to other audiences
- Take action to address social and reproductive injustices

**Table 1:** Principles of community-led research for reproductive justice in the SisterLove Georgia medication abortion project.

**Principle of community-led research for reproductive justice**	**Example from the SisterLove Georgia Medication Abortion project**
1) Center the marginalized community members most affected by SRH inequities as leaders of research	• The GAMA research study is led by community-based reproductive justice organization SisterLove, specifically bilingual Black and Latinx women leaders at SisterLove. • Overseen and guided by a Community Advisory Board including Black and Latinx women; community-based organizations serving Black and Latinx women; and Black and Latinx abortion providers and researchers.
2) Facilitate equitable, collaborative partnership through all phases of SRH research	• Conceptualization and Funding: The SisterLove team (spearheaded by bilinugal English and Spanish-speaking Black Latinx Sequoia Ayala) first conceived of the research study then applied for and secured funding by Society of Family Planning. Researchers at Emory University Center for Reproductive Health Research in the Southeast were approached as research partners with expertise in reproductive health data collection, analysis, and academic dissemination. • Data Collection and Analysis: After qualitative research training by Dr. Mosley, SisterLove led data collection and primary coding for the project and included Black and Latinx graduate student research assistants from Emory University, Georgia State, and other surrounding colleges and universities. • Dissemination: SisterLove leaders and staff members, student research assistants, community partners, and the academic researchers are co-authors and co-presenters at conferences; SisterLove led video and advocacy training development.
3) Honor multiple ways of knowing (experiential, cultural, empirical) for knowledge justice and cross-directional learning across the team	• White and academic team members were called in to understand and address biases and assumptions, while non-academic team members were supported to develop new qualitative research skills. • Black and Latinx Community Advisory Board members overseeing and guiding the entire research study included as co-authors and co-presenters at conferences. • Black and Latinx graduate student researchers, many of whom were bilingual, were supported to lead data collection and primary data coding at SisterLove. • All materials were available in English and Spanish including study instruments and dissemination materials such as the community newsletters, one-pagers, and the psychoeducational video.
4) Build on strengths (not deficits) within the community	• Interviews and focus groups emphasized both the facilitators and barriers of medication abortion in Black and Latinx communities, which illuminated creative solution for improving access. • Research project built on community trust in SisterLove and their existing research infrastructure, developed over decades of HIV and reproductive justice work with Black and Latinx communities in metro-Atlanta. This fostered buy-in from diverse recruitment partners, eagerness of community members to participate in the study despite the stigmatized topic, and rapid dissemination of findings and resources.
5) Implement the tenets of reproductive justice including structural-level analysis and the human rights framework	• Interview and focus group guides asked about the federal, state, and clinic-level policy environment surrounding medication abortion for Black and Latinx women, including abortion restrictions in Georgia such as the pending 6-week gestational age limit, 24 hour waiting period, and in-person dispensing requirements. • Interviews and focus groups explored intersectionality and medication abortion including how racism, immigration status, income, and gender intersect to create barriers to care
6) Prioritize disseminating useful findings to community members first then to other audiences	• The project began with a Community Kick-Off event, then continuously communicated about study activities and findings through a quarterly newsletter sent to all community members in the SisterLove communication networks. • The team developed a psychoeducational video about medication abortion designed for Black and Latinx women and available in English and Spanish with subtitles. The video addresses the most common misperceptions and misunderstandings about medication abortion as revealed during the study. • The team developed a Community and Clinical Advocacy Training for community members and clinicians who want to learn more about medication abortion and how to support Black and Latinx people to access those services.
7) Take action to address social and reproductive injustices	• Findings informed a psychoeducational video to improve knowledge about medication abortion to counteract low awareness of medication abortion and poor access to comprehensive sex education. • Findings about improvements abortion providers can make to increase patient-centered care for Black and Latinx patients (i.e., addressing bias, improving structural competency, increasing diversity of providers) were shared with clinical and public health audiences. • Findings were used in immediately during litigation against Georgia's HB 481, the state-level abortion restriction at 6 weeks, that was led by SisterSong, Feminist Women's Health Center, and others; for amicus briefs to the Supreme Court cases on abortion access (e.g., *Dobbs v. Jackson*); and during legislative debates against Georgia's SB 456, a proposed but defeated ban on telemedicine for medication abortion.

## Case study: The Georgia Medication Abortion project by SisterLove

### Study overview

We offer the community-led Georgia Medication Abortion (GAMA) Project from 2018–2022 as a case study to demonstrate these principles. The study was first conceptualized by bilingual, Black, and Latinx co-author Sequoia Ayala at SisterLove—an HIV and reproductive justice organization focused on women affected by HIV in metro-Atlanta since 1989. Ayala had observed disproportionate barriers to medication abortion for Black women and saw the need to investigate medication abortion perceptions and experiences among Black and other women of color. Meanwhile, the Society of Family Planning Research Fund had opened a medication abortion funding opportunity, to which SisterLove applied. With great excitement about this novel community-led approach, the Society of Family Planning invited SisterLove to submit a full proposal, but encouraged them to partner with a local academic research center to increase rigor of the proposed research methods. SisterLove intentionally selected Emory University's Center for Reproductive Health Research in the Southeast (RISE) given the University's positive research partnership record with SisterLove and given RISE's emphasis on reproductive health equity research, practice, and policy. Specifically, the RISE team included two monolingual, White researchers (Elizabeth Mosley and Kelli Hall) and one monolingual Black clinician-researcher and abortion provider (Tiffany Hailstorks). Collaboratively, the SisterLove and RISE team proposed a mixed methods study to explore Black and Latinx women's perceptions and experiences with medication abortion in Georgia. Our proposal was funded and became the first community-led research study by the Society of Family Planning. Notably, all Society of Family Planning funding applications now require explicit attention to community engagement, partnership, and dissemination.

The SisterLove-RISE team hosted a community kick-off event then organized a Community Advisory Board (CAB) with Black and Latinx women, researchers specializing in Black and Latinx reproductive health (including co-author Natalie Hernandez) Black and Latinx community-based organizations, abortion providers and clinic administrators (including co-author Kwajelyn Jackson), and religious leaders. This CAB helped develop data collection instruments; recruit participants; interpret preliminary results; and disseminate findings at conferences, in manuscripts, at clinics, and in community. From April 2019 to December 2020, the team conducted 10 key informant interviews with abortion providers (broadly defined to include physicians, nurses, receptionists, educators, administrators, and abortion funds) and 10 with leaders of Black and Latinx community-based organizations; 32 in-depth interviews with Black and Latinx women, and 6 focus groups with Black and Latinx women. The abortion provider interviews covered experiences with Black and Latinx abortion patients, their abortion method preferences and choices, and the facilitators and challenges of access. The Black and Latinx community-based organization interviews covered perspectives on abortion, awareness of medication abortion, and perceived barriers and facilitators of abortion access for their Black and Latinx clients. The in-depth interviews with Black and Latinx women covered awareness, knowledge, perspectives, and personal experiences with medication abortion as well as suggested solutions for improving access. Finally, the focus groups explored public attitudes toward medication abortion. Interviews and focus groups were conducted, transcribed, and analyzed by Black and/or Latinx Graduate Research Assistants at SisterLove with training and guidance from RISE researchers. Thematic analysis utilized memo-ing, coding, matrices, and group discussion to connect across codes, look for group differences (e.g., between Black and Latinx women, between patients and providers), and develop overarching themes.

### Main findings from the Georgia Medication Abortion project

The SisterLove-RISE team identified four overarching themes about medication abortion among Black and Latinx women. First, *at the socio-cultural level, intersectional oppression, intersectional stigma, and medical exploitation of Black and Latinx communities lead to systemic barriers*. For example, participants shared how population control and medical experimentation on Black and Latinx communities has led to distrust and fear of taking medication abortion pills, particularly from White providers. To address these barriers at the socio-cultural level, participants suggested medication abortion social marketing campaigns and story-sharing. Second, *at the policy level, participants described how policies from the federal (e.g., Hyde amendment), state (e.g., Georgia's impending 6-week gestational age limit), and institutional level (e.g., in-clinic medication abortion requirements) disproportionately affect Black and Latinx women*. They emphasized the importance of policy advocacy and political engagement to improve policies at the national and state level, while encouraging clinics to center Black and Latinx patients in their protocols for patient-centered, equitable access to care. Third, *at the clinic and provider levels, participants described factors that inadvertently made abortion services less accessible or less patient-centered for Black and Latinx patients*. This included lack of representative (Black, Latinx, Spanish-speaking) abortion providers and clinic staff, provider knowledge gaps in structural competency, implicit provider bias (e.g., recommending surgical abortion and not offering medication abortion to Black patients because of adherence concerns), and high costs of medication abortion. Solutions included diversifying the abortion clinic team, flexible scheduling, sliding scale fees, holistic reproductive justice abortion funds, and de-medicalization including at-home telemedicine for medication abortion. Finally, *at the individual-level, Black and Latinx women faced numerous barriers including “lack of information…education… money…fear of…shaming…lack of public transportation…fear of [immigration] raids.”* In other words, the common stigma-related barriers to medication abortion are compounded by “*all the things that make life difficult”* for Black and Latinx communities. Participants offered inspiring solutions including social support (e.g., from one's own network and from abortion doulas, holistic reproductive justice abortion funds) and word-of-mouth story-telling by Black and Latinx people about medication abortion.

### Community-based and policy-focused dissemination of findings

**Figure 1:**
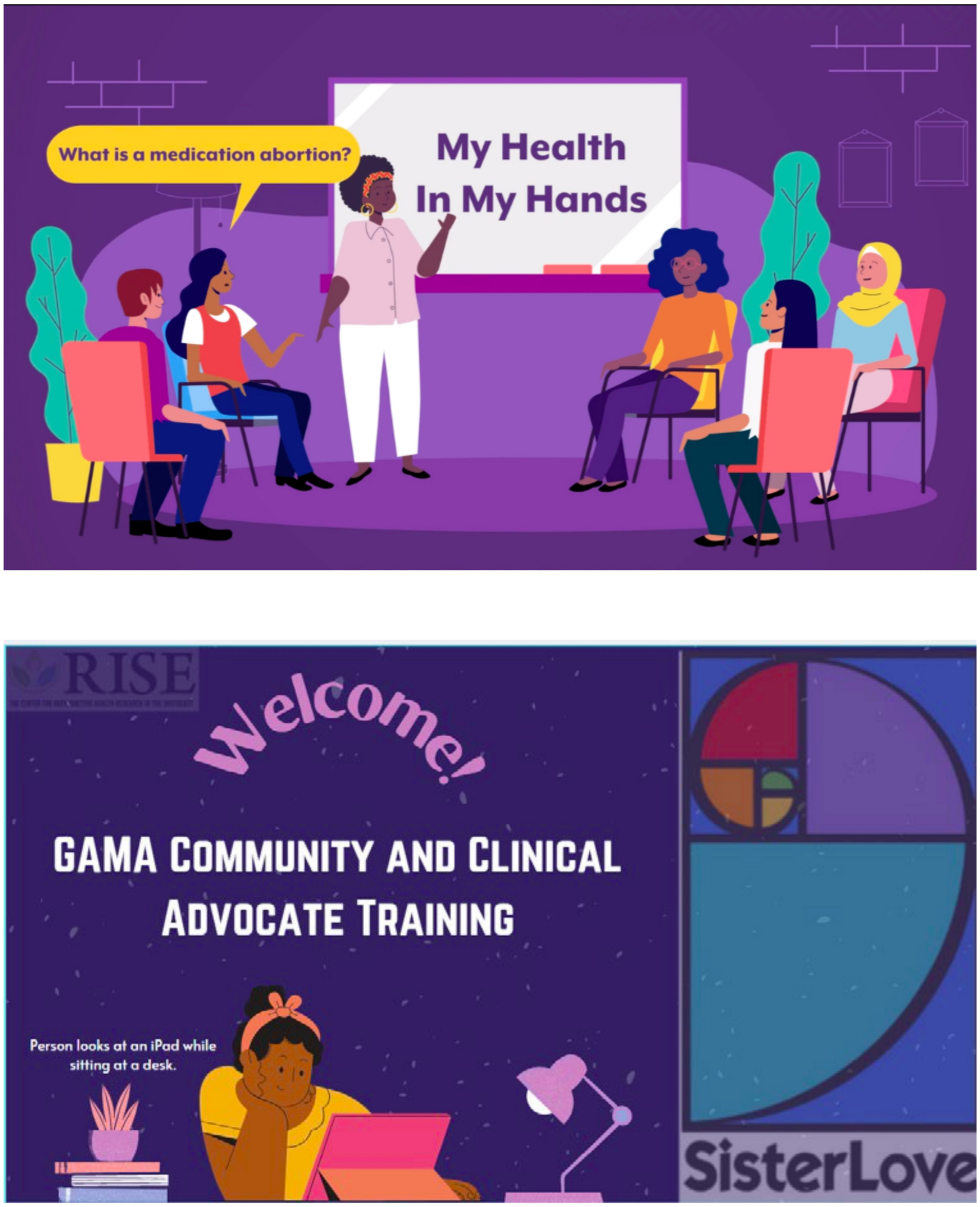
My health in my hands medication abortion psychoeducational video and the community-clinical advocacy training.

The study's main findings have been presented at conferences, as published abstracts, and are currently under review with peer-reviewed journals (32, 33). More importantly, the findings have been disseminated back to Black and Latinx communities in metro-Atlanta and beyond through quarterly newsletters, fact sheets about medication abortion, a psychoeducational video, and a community-clinical advocacy training. To address low levels of awareness and knowledge about medication abortion, the team developed a psychoeducational video about medication abortion designed specifically for and by Black and Latinx women (see Figure 1). The 2.5 min video is narrated by Damaris, a Black health educator at SisterLove, and set at the MotherHouse—a home-turned-office and clinic in Southwest Atlanta shared by SisterLove and the SisterSong Women of Color Reproductive Justice Collective. The video, available in English and Spanish, provides information about how medication abortion works and where to access it, addresses common misconceptions we heard in the GAMA study, and explains the policies regulating medication abortion. The psychoeducational video has been pilot tested with 850 people, which showed the video significantly increases knowledge and reduces racial/ethnic disparities in medication abortion knowledge. An ongoing evaluation with a representative sample of Black and Latinx women is being conducted, once updates are made for changes in telemedicine for abortion and the impending Supreme Court decision. After that, the video will be widely disseminated at abortion clinics, at reproductive health and justice organizations, on social media, and in sex education programs.

The team also developed a community-clinical advocacy training for community-based organizations and clinical providers, who want to learn more about medication abortion and support Black and Latinx community members to safely access medication abortion (see Figure 1). This webinar-style training is self-paced and provides more detailed instruction about medication abortion, policies regulating medication abortion, and how to connect people to medication abortion care. It is currently being pilot tested at SisterLove and will then be disseminated to Black and Latinx serving clinics and community-based organizations nationally.

Findings from GAMA were also leveraged for reproductive health policy advocacy including in amicus briefs for Supreme Court cases on abortion restriction well as for testimony and legislator education about state-level bans on telemedicine for abortion. Qualitative data from GAMA were included in the *Dobbs v. Jackson* case to emphasize how abortion restrictions disproportionate and unjustly affect Black and Latinx patients, as well as to catalog the numerous barriers they already face when trying to access abortion care. In 2022, conservative Georgia legislators introduced a ban on telemedicine for abortion (SB 456). This came shortly after the U.S. Food and Drug Administration had eliminated in-person physician dispensing of mifepristone and in the wake of COVID-19, when other in-person clinical requirements had been loosened. GAMA researcher Elizabeth Mosley testified twice against the bill, and she mentored GAMA Graduate Student Researcher Priya Shah to develop a legislator education toolkit about the safety of telemedicine for medication abortion, which was distributed to all Georgia legislators. SB 456 did not pass.

### Strengths and challenges of SisterLove's Georgia Medication Abortion project

Notable strengths of this community-led reproductive justice approach to research include:

- Emphasis on community-based dissemination leading to timely, impactful community resources.
- Policy relevant data collected by advocacy-focused community organizations are efficiently and effectively leveraged for community organizing and policy advocacy.
- Capacity building for research within the communities most impacted by sexual and reproductive health disparities.
- Capacity building for community engagement, cultural humility, and structural competency among academic researchers.
- Novel findings imperative for improving reproductive health outcomes and equity, which are obscured by traditional research methods.

Despite these successes, the project faced structural challenges including:

- Underfunding of community-led and community-engaged research by federal, foundation, and other funders.
- Project team member turnover for both the community and academic partners.
- Institutional barriers to community-academic research collaboration.
- Devaluation of community-engaged research at leading family planning peer-reviewed journals.

SisterLove, like all community-based and reproductive justice organizations, is structurally under-funded. For example, the GAMA project originally proposed a state-wide survey in addition to the qualitative study—all in 2 years. With a budget of only $300,000, our team lacked the staff capacity and resources to execute a full mixed methods study in that timeline. Ultimately, we prioritized the psychoeducational video and community-clinical advocacy training over the quantitative survey, because this was of greatest importance to community members. Another major challenge was team turnover both at SisterLove and RISE. Institutional barriers to community-academic collaborations included requirement of IRB approval before any funding is released, academic professional advancement requiring peer-reviewed and first-author publications in high impact journals, and relatively lower capacity at community-based organizations to be the primary administrator of large research grants. Finally, as the team has turned toward academic dissemination *via* publishing in peer-reviewed journals, we met startling resistance from some SRH colleagues. When submitting our results to leading SRH journals, we mostly received constructive feedback and encouragement. However, there were still reviewers who called community-led research “less rigorous” and accused us of prejudice against white abortion providers.

## Discussion

Community-led reproductive justice research offers innovative and transformational methods for truly advancing SRH. This approach is needed in the United States now more than ever given the impending overturn of *Roe v. Wade* and with it the federal protection of abortion rights. States controlled by conservative governments, such as Georgia, will severely restrict access to abortion (i.e., at 6 weeks or possibly worse). This will undoubtedly affect Black, Latinx, and low-income communities most. The GAMA project led by SisterLove offers an important example of how community-led reproductive justice research can produce high-quality scientific evidence, disseminate benefits back to affected communities, and advocate against unjust reproductive healthcare restrictions. Yet existing funding, research administration, and publishing systems are not designed for community-led research for reproductive justice. Private foundations, federal institutions (e.g., National Institutes of Health), universities, professional organizations (e.g., Society of Family Planning, American College of Obstetricians and Gynecologists), and peer-reviewed journals will require considerable evolution to promote research and SRH equity.

## Data availability statement

De-identified qualitative datasets generated for this study can be accessed by contacting SisterLove, Inc. at research@sisterlove.org for an application. Requests to access the datasets should be directed to research@sisterlove.org.

## Ethics statement

The studies involving human participants were reviewed and approved by Emory University Institutional Review Board. The participants provided their verbal informed consent to participate in this study.

## Author contributions

EM wrote the first draft of this manuscript. SA, ZJ, TH, DD, NH, KJ, IH, and KH provided revisions on the manuscript. SA originally conceived of the Georgia Medication Abortion project. DD, KH, SA, EM, and IH secured original and ongoing funding for the Georgia Medication Abortion project. SA, ZJ, and IH coordinated research for the Georgia Medication Abortion project including all data collection and analysis. KJ and NH led the Community Advisory Board including instrument development, recruitment, and dissemination. All authors contributed to the article and approved the submitted version.

## Funding

The Georgia Medication Abortion Project is funded by the Society of Family Planning Research Fund, an Anonymous Foundation, and through the Center for Reproductive Health Research in the Southeast (RISE) including support from the Collaborative for Gender and Reproductive Equity, a sponsored project of Rockefeller Philanthropy Advisors.

## Conflict of interest

Author EM works as a consultant for SisterLove, Inc. providing community-based qualitative research methods training. Authors SA, ZJ, DD, and IH are employed by SisterLove, Inc. The remaining authors declare that the research was conducted in the absence of any commercial or financial relationships that could be construed as a potential conflict of interest. The handling editor MM declared a past co-authorship with the author SA.

## Publisher's note

All claims expressed in this article are solely those of the authors and do not necessarily represent those of their affiliated organizations, or those of the publisher, the editors and the reviewers. Any product that may be evaluated in this article, or claim that may be made by its manufacturer, is not guaranteed or endorsed by the publisher.

## Footnotes

^1^Founding Mothers of reproductive justice also include Toni M. Bond Leonard, Reverend Alma Crawford, Evelyn S. Field, Terri James, Bisola Marignay, Cassandra McConnell, Cynthia Newbille, Loretta Ross, Elizabeth Terry, ‘Able' Mable Thomas, Winnette P. Willis, and Kim Youngblood.
